# A Generic Design of Driver Drowsiness and Stress Recognition Using MOGA Optimized Deep MKL-SVM

**DOI:** 10.3390/s20051474

**Published:** 2020-03-07

**Authors:** Kwok Tai Chui, Miltiadis D. Lytras, Ryan Wen Liu

**Affiliations:** 1 Department of Technology, School of Science and Technology, The Open University of Hong Kong, Hong Kong; 2 School of Business & Economics, Deree College—The American College of Greece, 153-42 Athens, Greece; mlytras@acg.edu; 3 Effat College of Engineering, Effat University, Jeddah P.O. Box 34689, Saudi Arabia; 4 Hubei Key Laboratory of Inland Shipping Technology, School of Navigation, Wuhan University of Technology, Wuhan 430063, China; wenliu@whut.edu.cn

**Keywords:** at-risk driving, deep support vector machine, driver drowsiness, driver stress, multi-objective genetic algorithm, multiple kernel learning

## Abstract

Driver drowsiness and stress are major causes of traffic deaths and injuries, which ultimately wreak havoc on world economic loss. Researchers are in full swing to develop various algorithms for both drowsiness and stress recognition. In contrast to existing works, this paper proposes a generic model using multiple-objective genetic algorithm optimized deep multiple kernel learning support vector machine that is capable to recognize both driver drowsiness and stress. This algorithm simplifies the research formulations and model complexity that one model fits two applications. Results reveal that the proposed algorithm achieves an average sensitivity of 99%, specificity of 98.3% and area under the receiver operating characteristic curve (AUC) of 97.1% for driver drowsiness recognition. For driver stress recognition, the best performance is yielded with average sensitivity of 98.7%, specificity of 98.4% and AUC of 96.9%. Analysis also indicates that the proposed algorithm using multiple-objective genetic algorithm has better performance compared to the grid search method. Multiple kernel learning enhances the performance significantly compared to single typical kernel. Compared with existing works, the proposed algorithm not only achieves higher accuracy but also addressing the typical issues of dataset in simulated environment, no cross-validation and unreliable measurement stability of input signals.

## 1. Introduction and Literature Review

The World Health Organization (WHO) has reported that the annual road traffic deaths and injuries remain unacceptably high as 1.35 million and 50 million respectively [1]. It has highlighted the road traffic is the 8th leading cause of death for people of all ages. More important, it ranks number one when it comes to the age group of 5–29 years old, which can wreak havoc on economic and social development. To be transformed into monetary measure, it accounts for 3% loss (equivalent to 2400 billion USD) in world gross domestic product (GDP). Injuries include minor cuts, whiplash, bruises to broken limbs, paralysis and spinal injuries. The report from the American Automobile Association (AAA) concluded that the poor driving behaviors leads to over 55% of fatal crashes [2]. With the ever-growing number of cars, the leading cause of death will soar from the 9th to the 7th position by 2030 if no action and prevention are carried out. In this paper, the issues of driver drowsiness and stress will be addressed.

Driver drowsiness is about the sleepiness of drivers that is an undesired condition in real-world driving. Surveys revealed that more than half of adult drivers felt sleepy while driving and even more severe that about 30% of them fell asleep [3]. This high prevalence can further estimate that drivers could experience accidents (affect or being influenced attributable to drowsy driving). It is worth mentioning that driver drowsiness is differed from driver fatigue [4]. Drivers drive unconsciously in the former condition but are with a conscious status in latter condition. In reality, people are usually fatigued in today’s fast paced world.

Stress is body’s way to react any kind of threat, challenge and demand. Research has revealed that stress has played a crucial role in adapting to driving and making decision [5]. The primary sources for driver stress are congestion and adverse driving condition as well as time pressure [6]. The stress can lead to poor and dangerous driving behaviors, for instance, flashing high beams, eliciting anger in drivers, road rage and aggressive driving, which are major causes of road traffic accidents [7]. Various recent studies have been carried out on the investigation between psychological factors and driving behaviors. In [8], results revealed that anger leads to stress, which is reflecting in the form of aggressive and negative cognitive driving behavior. On the other hand, research argued that risky drivers generally exhibited more antisocial and substance misuse, reward sensitive personality features and sensation seeking [9]. In addition, traffic penalties reported by public transport drivers are preceded by individual factors, personality and work-related when combined with driving anger. It may enhance negative results on traffic sanctions given they are preceded by risky road behaviors and affect overall road safety [10].

Repeated exposure of stressful conditions affects drivers’ daily life and drivers have higher risks in suffering from stress-related health problems, for instance, accelerated aging [11], depression and anxiety [12], type 2 diabetes [13], asthma [14] and cardiovascular diseases [15]. These have indicated that driver stress can lead to long-term health issues.

### 1.1. Literature Review

Various research works have been carried out in driver drowsiness and driver fatigue detection. We believe that this paper is the first work that fully considers a generic model that can apply to recognize both driver drowsiness and stress. In this subsection, the literature review is divided into two parts, which firstly present the latest works on driver drowsiness recognition.

#### 1.1.1. Existing Works of Driver Drowsiness Recognition

Typical algorithms for driver drowsiness recognition were based on three types of inputs: (i) the biometric-signal-based approach [16,17,18,19]; (ii) the vehicle-based approach [20,21,22,23] and (iii) the image-based approach [24,25,26,27]. Approach (i) is intrusive whereas approaches (ii) and (iii) are non-intrusive.

The first approach is illustrated as follows. In [16], driver drowsiness recognition was based on the input of respiratory signal, which measurement requires the tracking of the displacements of the diaphragm, abdominal and rib cage. A threshold was derived by analyzing the respiratory rate variability of the training dataset. The first work to adopt the electrocardiogram (ECG) signal was presented in [17], which is differed from traditional works that relied on partial information of the ECG signal, R wave or heart rate variability (HRV). The feature vector was formulated by cross-correlation coefficient between ECG signals and the classification problem was modeled by support vector machine (SVM). Another biometric-signal-based approach includes the electroencephalogram (EEG) signal, examples can be referred to [18] and [19] for the SVM model and long short-term memory (LSTM) model respectively.

When it comes to the vehicle-based approach, a lightweight threshold-based algorithm was proposed to analyze the status of drivers, with the continuous input of steering wheel angle [20]. With the same input, multilevel ordered logit model was implemented [21]. Some works utilized more measurements as inputs for driver drowsiness recognition. For instance, in [22], besides the steering wheel angle, pedal input, vehicle speed and acceleration were selected as features for classification model based on the dynamic Bayesian network. The deviation from the current lane could also be a useful indicator of driver drowsiness, as verified using an exponentially weighted moving average [23].

The third approach is based on the images of drivers, specifically the images are retrieved from continual video frames recorded by an in-vehicle camera. The textual and landmark information of the drivers’ face were collected as inputs of the driver drowsiness recognition algorithm, implemented by the deep belief network [24]. Researchers in [25] constructed the features based on the detection of the face, eyes, nose and mouth of the drivers. A threshold was determined on the number of eye blinks per minute in order to deduce the percentage of drowsiness level. Mandal et al. proposed a fusion and reasoning method to measure the driver drowsiness, the following modules were included, head and shoulder detection, face detection based on the front view and oblique view analysis, eye detection based on Open Source Computer Vision Library (OpenCV) and Institute for Infocomm Research (I2R) as well as the eye openness estimation [26]. A multi-channel (3, 6 and 9 channels) second-order blind identification algorithm was proposed, which analyzed the yawn signals and eye blinks and yielded optimal thresholds for the drowsiness level [27].

#### 1.1.2. Existing Works of Driver Stress Recognition

When it comes to driver stress recognition, there are less research publications compared to driver drowsiness recognition. However, both are of equal importance as life threatening sources. The approach for driver stress recognition was mainly based on the biometric-signal-based approach. Khattak et al. carried out statistical analysis (Wilcoxon Signed rank test, *t*-test and analysis of variance (ANOVA)) on the driver stress based on drivers’ HRV [28]. Electrodermal activity (EDA) skin potential response (SPR) is another typical input for the driver stress detection, which an example was shown along with adaptive filtering and spike detection techniques [29]. Skin conductance and EEG served as inputs of the driver stress condition, which were further explored by the incremental association Markov blanket algorithm for feature extraction [30]. The least square support vector machine (LS-SVM) was applied to construct the classifier. In [31], researchers discussed the feasibility of the employment of HRV and photoplethysmogram (PPG) signal for driver stress recognition through ensemble learning of k-nearest neighbor (kNN), decision tree (DT) and linear discriminant analysis (LDA). Sparse Bayesian learning (SBL) and principal component analysis (PCA) were adopted to find out the optimal feature vector from a galvanic skin response (GSR), HRV and respiration [32]. Two kernel-based methods SVM and extreme learning machine (ELM) were applied and evaluated with typical kernels, sigmoid, radial basis function (RBF) and linear kernel.

It is noted that there were a few discussions on driver stress recognition via other approaches like steering wheel angle [33], steering wheel angle and road shape [34] and speech signal [35]. Besides, there is a recent work that detected the driver’s status among normal, stress, fatigue and drowsiness [36]. However, it has been limited by assuming only one status. Differing from the authors work, we perform drowsiness and stress recognition in parallel to each other so that drivers will have multiple statuses, awake/drowsy and stress level.

### 1.2. Research Gaps and Motivation

Various methods have been proposed for driver drowsiness recognition [16,17,18,19,20,21,22,23,24,25,26,27] and driver stress recognition [28,29,30,31,32,33,34,35]. Nonetheless, there exist several challenges and limitations that require further research.

- There is no generic model for driver drowsiness and stress recognition, which is one model suits two applications. Existing studies considered the formulation separately and required the implementation of different models to serve two applications. This may increase the complexity of the recognition system as well as include more input signals.
- The study in [37] analyzed the measurement reliability of several signals, ECG, EEG and video recording on real-world driving conditions (highway, urban and turning). EEG and video recording may not be reliable input signals as only 85% and 59% of the time the acquired signals are with good signal quality. The severe signal distortion in the rest of the period could alter the validity of the foundation of the problem formulations, which could be considered as misleading input and thus output. Therefore, EEG and video-based approaches experience challenges in signal acquisition.
- There was limited utilization of a full ECG signal as signal input [17], which most of the existing works were focusing on HRV, which is a single point of an ECG signal. An in-depth analysis could be made on driver drowsiness and stress recognition via ECG signal.
- Generally, shallow learning was adopted in existing works, except the discussion of a deep belief network in [24] for driver drowsiness recognition. Since there is room for improvement of accuracy of the existing classifiers, applying and investigating the deep learning technique could benefit on the classification performance.

To address the aforementioned challenges, the following measures are introduced in this paper:

- A generic model is proposed for the recognition of both driver drowsiness and stress.
- The high measurement reliability ECG signal is adopted as the input signal.
- The complete ECG signal is employed so that more representative features can be included in the feature extraction process.
- The deep learning approach is selected to build the classifier. It is noted that there is a consideration of deep learning with small samples, which implies that typical deep neural network is not appropriate.

### 1.3. Research Contributions

The key contributions of this paper are summarized as follows.

- A generic model using the multiple-objective genetic algorithm (MOGA) optimized deep multiple kernel learning support vector machine (D-MKL-SVM) is proposed for the recognition of driver drowsiness and stress detection.
- The ECG signal is newly applied for driver stress recognition in which the idea of using the ECG signal as an input was proposed in [17] for driver drowsiness recognition. The identical input signal and model serving two applications, i.e., driver stress and drowsiness recognition could reduce the complexity of the system theoretically and practically.
- Deep support vector machine is employed, which takes the advantage in small samples problem.
- The proposed generic model achieves highest accuracy for both driver drowsiness recognition and driver stress recognition compared to existing works. It also addresses typical issues of existing works, which are input signals of poor measurement stability, performance evaluation without cross-validation and collecting data using the simulated environment.

## 2. Materials and Methodology

In this section, the dataset for driver drowsiness and stress data was firstly presented. It was followed by data pre-processing techniques as well as beat segmentation for the ECG signal. Afterwards, the proposed generic model using MOGA optimized D-MKL-SVM would be discussed.

### 2.1. Driver Drowsiness and Stress Dataset

**Figure 1:**
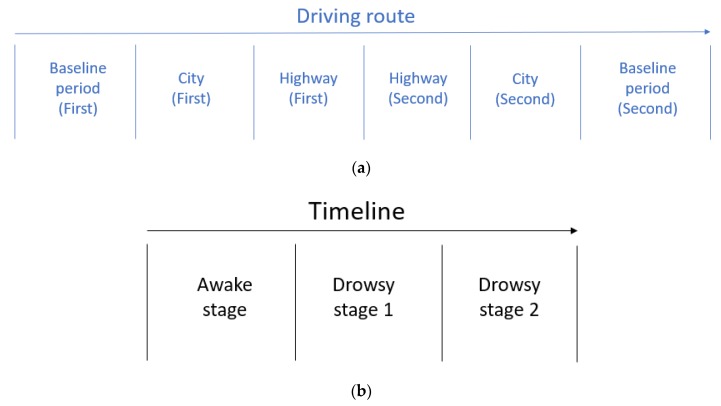
(**a**) Scenario setting in the Stress Recognition in Automobile Drivers Database and (**b**) the scenario setting in the cyclic alternating pattern (CAP) Sleep Database.

The dataset for driver drowsiness and stress data was retrieved from publicly available databases: the Stress Recognition in Automobile Drivers Database [38,39] and the cyclic alternating pattern (CAP) Sleep Database [39,40]. The first database includes 18 records of real-world driving in Boston, USA, which the route was started from the first baseline period, to the first city, to the first highway, to the second highway, to the second city and ended with the second baseline period, which is summarized in Figure 1a. The experiment was conducted in mid-morning or mid-afternoon to avoid heavy road traffic. Each participant was at rest (eyes closed and sitting inside the vehicle) for 15 minutes before and after the driving. This period of data provides a baseline of the participant. The medium stress environment was set-up by highway driving between a toll preceding the off-ramp and at the on-ramp. The high stress environment was side street and main street driving environment with a winding, narrow lamp. The ECG signal of the drivers were continually monitored and collected. The duration of records ranged from 50 to 90 min. Three stress levels: (i) high stress level; (ii) medium stress level and (iii) low stress level; were defined. To avoid the effect of traffic condition, the experiment was carrying out at mid-morning or mid-afternoon.

The second database had 108 records from sleep centers. These records contained six categories of sleep stages (i) awake stage; (ii) sleep stage 1; (iii) sleep stage 2; (iv) sleep stage 3; (v) sleep stage 4 and (vi) rapid eye movement (REM) stage. Only sleep stage 1 and sleep stage 2 were selected, which is drowsy stage 1 and drowsy stage 2 because they were the immediate stages after awake stage, which the timeline is shown in Figure 1b.

### 2.2. Data Pre-Processing

The data pre-processing of the dataset aimed at segmenting ECG beat into individual samples. We have adopted an existing Tompkins’ method [41,42] as it achieves favorable performance in ECG beat segmentation. The major steps involved are dc offset elimination, digital band-pass filter, derivative filter, signal squaring, sliding window integration and locating Q, R and S waves. The detail was omitted as this was not the focus of this paper.

There are three classes in each of the driver drowsiness dataset and driver stress dataset. Their corresponding number of samples is tabulated in Table 1 after ECG beat segmentation. Totally, the driver drowsiness dataset and driver stress dataset contain 131,500 and 76,200 samples respectively.

### 2.3. Generic Model Using MOGA Optimized D-MKL-SVM

**Figure 2:**
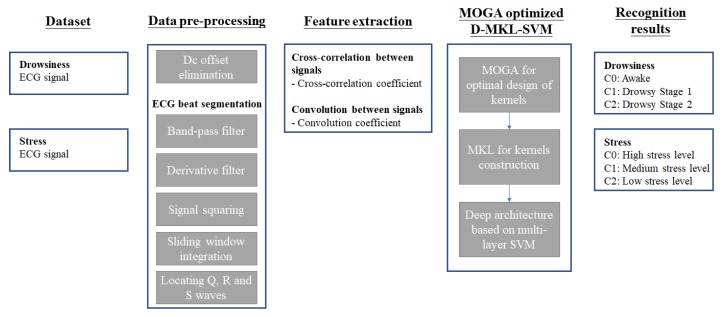
General flow of the proposed generic model multiple-objective genetic algorithm (MOGA) optimized deep multiple kernel learning support vector machine (D-MKL-SVM).

We have proposed a generic model using MOGA optimized D-MKL-SVM. Figure 2 shows the general flow of the model. Dataset and data pre-processing were discussed in Section 2.1 and Section 2.2 respectively. For feature extraction, the rationale is explained as follows. Previous works had revealed that HRV or R waves of ECG signals could serve as an important feature for driver drowsiness recognition. In this paper, the consideration was further extended to a full utilization of the ECG signal. Cross-correlation and convolution techniques were applied between every two samples (as defined in Section 2.2), which resulted in cross-correlation coefficients and convolution coefficient and serving as feature inputs of driver drowsiness and stress data.

Cross-correlation between two ECG signals X_1_ and X_2_ with length of N = 100 (zero-padding when len{X}<100) is given in Equation (1).

(1) $${\mathrm{XCorr}}_{X_{1},X_{2}}\left[k\right]=\{\begin{array}{ll} \overset{N-1}{\underset{n=k}{\sum }}X_{1}\left[n\right]X_{2}\left[n-k\right], & k\geq 0\\ \overset{N-\left|k\right|-1}{\underset{n=0}{\sum }}X_{1}\left[n\right]X_{2}\left[n-k\right], & k<0 \end{array}$$

Convolution between X_1_ and X_2_ can be calculated by:

(2) $${\mathrm{Con}}_{X_{1},X_{2}}\left[k\right]=\overset{N-1}{\underset{k=0}{\sum }}X_{1}\left[n\right]X_{2}\left[n-k\right]$$

The model for driver drowsiness and stress recognition is built based on deep multi-layer SVM architecture. Generally, it consists of multiple hidden layers of SVM and an output layer of SVM. Compared to other deep learning architectures, multi-layer SVM takes several advantages like (i) the output layer SVM has strong regularization power to avoid over-fitting; (ii) able to handle problem of very large input vectors and few training samples and (iii) the design of kernel functions is more flexible.

**Figure 3:**
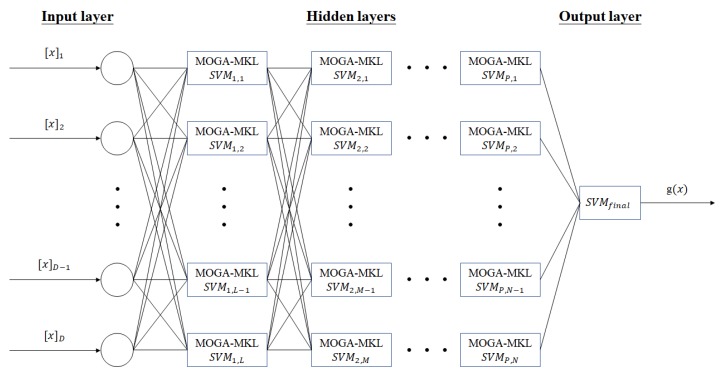
Architecture of MOGA optimized D-MKL-SVM.

The deep multi-layer SVM is structured as in Figure 3. Assume D, L, M and N are integers. Here the number of hidden layers had not been fixed, which is a general form of architecture. In this paper, experimental results indicate that the optimal setting for driver drowsiness and driver stress recognition is deep three-layered SVM and deep four-layered SVM respectively. In each of the SVM, the kernel function is customized by multiple kernel learning (MKL), which weighting factors $\omega_{i}=\left[0,1\right]\;\forall i=1,\ldots ,N_{k}$ where there is N_k_ kernels, are optimally designed by MOGA.

The kernel function of every SVM is designed by the combinations of typical kernels, linear kernel, RBF kernel, polynomial kernel and sigmoid kernel. The classifier can achieve better performance by taking the advantages from each kernel. Four kernel properties are applied for joining these kernels. Weighting factors are also introduced between kernels and optimally solved by MOGA. It is worth mentioning that, each SVM in multi-layer SVM architecture may result in different sets of optimal weighting factors. The kernel properties are presented as follows [43]. Assume there are two Mercer’s kernel $K_{i}\left(x_{1},x_{2}\right):\chi \cdot \chi \to R$ and $K_{j}\left(x_{1},x_{2}\right):\chi \cdot \chi \to R$, $K_{e}\left(x_{1},x_{2}\right)$ is the resultant Mercer’s kernel. It is trivial to derive that joining properties between P_1_, P_2_, P_3_ and P_4_ in Equations (3)–(6) is also governed by Mercer’s theorem.

(3) $$P_{1}:K_{e}\left(x_{1},x_{2}\right)=K_{i}\left(x_{1},x_{2}\right)+K_{j}\left(x_{1},x_{2}\right)$$

(4) $$P_{2}:K_{e}\left(x_{1},x_{2}\right)=b\cdot K_{i}\left(x_{1},x_{2}\right)$$

(5) $$P_{3}:K_{e}\left(x_{1},x_{2}\right)=K_{i}\left(x_{1},x_{2}\right)+b$$

(6) $$P_{4}:K_{e}\left(x_{1},x_{2}\right)=K_{i}\left(x_{1},x_{2}\right)\cdot K_{j}\left(x_{1},x_{2}\right)$$

The optimal design of kernel functions for each SVM is considered as a multi-objective optimization (MOO) problem, which is solved by MOGA. MOO is practically important because daily life applications are generally with multi-objective and those objectives are conflicted with each other [44,45]. Traditional single objective optimization often normalizes and combines objectives into single objective; whereas MOO provides trade-off optimal solutions, and these compromise of the solutions and can increase the satisfaction of the decision-makers. In addition, MOO is characterized by different search spaces, multiple objectives and cardinalities-optimal solutions [46]. Here are quick review of definitions of important concepts: (i) Objective space is defined as the multidimensional space of the objective functions; (ii) Pareto optimal solution is defined as the optimal solution in the objective space and (iii) Pareto front is defined as the set of Pareto optimal solutions.

Attributed to multiple conflicting objectives, there exists a challenge in obtaining single optimal objective. As a result, the technique of domination was introduced. Consider a minimization problem of the N-objective, we say candidate solution a is dominated by another candidate solution b if and only if function values of a is partially less than b, its mathematical expression is:

(7) $$\{\begin{array}{ll} f_{n}\left(a\right)\geq f_{n}\left(b\right), & \forall n=1,2,\ldots ,N\\ f_{n}\left(a\right)>f_{n}\left(b\right), & \exists n=1,2,\ldots ,N \end{array}$$

**Figure 4:**
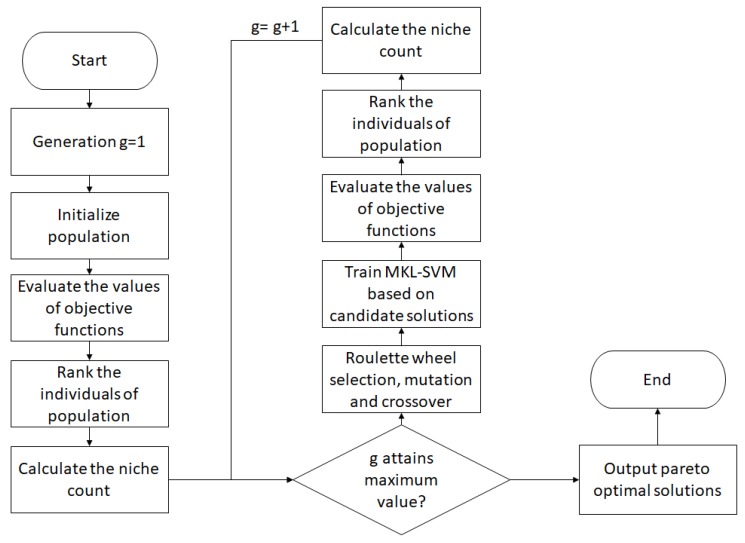
MOGA process for the optimal design of MKL-SVM.

Hence, non-dominated Pareto optimal solutions are desired. The flow of the MOGA for the optimal design of MKL for each SVM is shown in Figure 4. The key steps are summarized:

- Initialize the values of objective function as well as the population size.
- Based on the first step, compute the values of the objective function for all individuals within the population. This creates a list of values.
- Rank the individuals based on the list of values in Step (ii).
- For a defined population size, the nature of stochastic selection errors governs the convergency of the population, which depends on a small group of Pareto optimal solutions, rather than all optimal solutions.
- Lengthen the distance between Pareto optimal solutions along the axis of objective functions. This could increase the diversity of the population in order to lower the tendency of the convergence to small group solutions. Thus, the niche count is utilized and defined.
- Generate a new offspring.
- Evaluate the values of objective functions.
- Calculate ranks assignment and niche count repeatedly in the new offspring.
- MOGA can be terminated in two situations: (i) the output reaches the Pareto front; or (ii) generation (number of iterations) reaches the maximum number of generations.

The MOO for driver drowsiness and stress recognition is formulated as follows, with objectives O_1_, O_2_ and O_3_:

(8) $$O_{1}:\;\max\;\overset{N}{\underset{i=1}{\sum }}\alpha_{i}-0.5\overset{N}{\underset{i=1}{\sum }}\overset{N}{\underset{j=1}{\sum }}\alpha_{i}\alpha_{j}y_{i}y_{j}K_{e}\left(x_{i},x_{j}\right)$$

(9) $$O_{2}:\;\max\;\mathrm{TN}/N_{n}$$

(10) $$O_{3}:\;\max\;\mathrm{TP}/N_{p}$$

where O_1_ aims at maximizing the margin, defining $\alpha_{i}$ as the Lagrange multiplier, $y_{i}\in \left\{-1,+1\right\}$ as the output class and $K_{e}\left(x_{i},x_{j}\right)$ is the resultant Mercer’s kernel defined by applying kernel properties in Equations (3)–(6) on typical kernels: linear kernel, RBF kernel, polynomial kernel and sigmoid kernel. O_2_ maximizes the specificity, which is the ratio of true negative (TN) and number of negative samples (N_n_). O_3_ maximizes the sensitivity, which is the ratio of true positive (TP) and number of positive samples (N_p_).

The binary classifier SVM is extended to multi-class SVM by the 1-against-1 method as it outperforms the one-against-all approach in general applications [47,48,49]. When it comes to performance evaluation, 10-fold cross-validation is selected [43,49,50].

## 3. Analysis and Results

Various scenarios were analyzed to reveal the effectiveness of proposed generic model using MOGA optimized D-MKL-SVM for driver drowsiness and stress recognition. Four parts would be discussed in detail: (i) the performance of the proposed generic model MOGA optimized D-MKL-SVM was evaluated; (ii) study of the effectiveness of MOGA comparing with pure D-MKL-SVM; (iii) comparing the performance between MKL and single typical kernel and (iv) comparing the performance of proposed algorithm and related works.

### 3.1. Performance Evaluation of MOGA Optimized D-MKL-SVM

**Figure 5:**
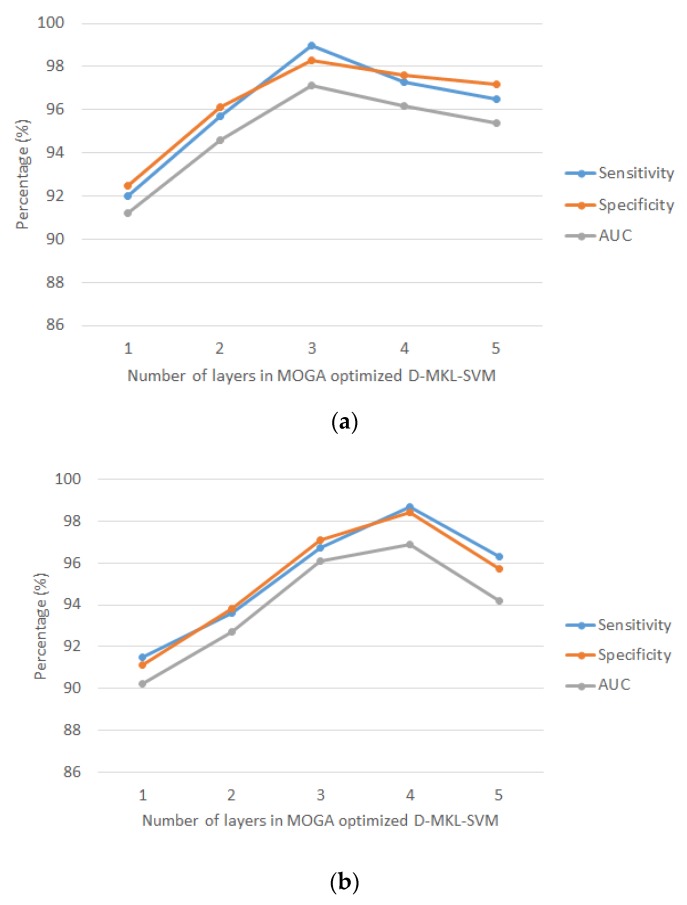
Average sensitivity, specificity and area under the receiver operating characteristic curve (AUC) versus number of layers in MOGA optimized D-MKL-SVM under 10-fold cross-validation: (**a**) driver drowsiness recognition and (**b**) driver stress recognition.

The evaluation criteria for driver drowsiness and stress recognition via the MOGA optimized D-MKL-SVM algorithm are the specificity, sensitivity and area under the receiver operating characteristic curve (AUC). Figure 5 shows the average of sensitivity, specificity and AUC (based on 10-fold cross-validation) of driver drowsiness and driver stress versus the number of layers in MOGA optimized D-MKL-SVM. As a trade-off to computation time, the number of layers was limited to 5.

**Figure 6:**
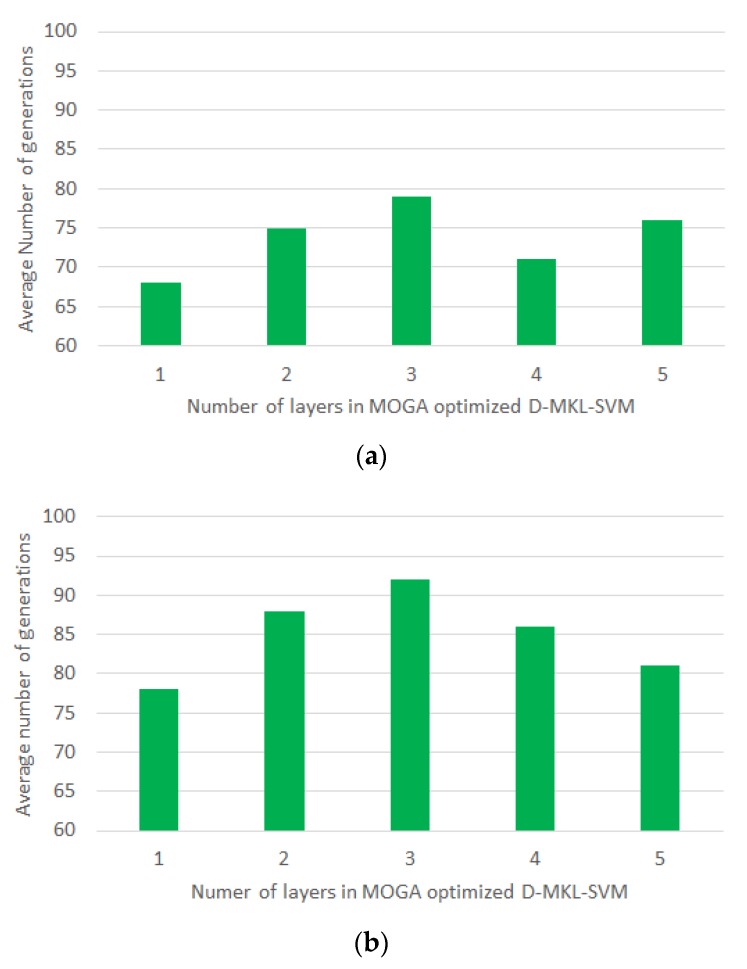
Average number of generations versus the number of layers in MOGA optimized D-MKL-SVM under 10-fold cross-validation: (**a**) driver drowsiness recognition and (**b**) driver stress recognition.

Results revealed that the proposed algorithm achieved favorable performance in average sensitivity, specificity and AUC in both driver drowsiness and stress recognition. For driver drowsiness recognition, the best performance was obtained using deep three-layered SVM, with average sensitivity of 99%, specificity of 98.3% and AUC of 97.1%. For driver stress recognition, the best performance was yielded when deep four-layered SVM was adopted, with average sensitivity of 98.7%, specificity of 98.4% and AUC of 96.9%. On the other hand, the average number of generations (rounded to nearest integer) in each layer for driver drowsiness and stress recognition is shown in Figure 6. As a result, the proposed approach was generic, which suited both applications.

### 3.2. Study on the Benefits of MOGA

For parameter selection, grid search is a typical method that would carry out a simple trial and error on the values parameters, given a range and certain step size between successive test values. The grid search method requires one to test all the scenarios with the step size that requires excessive computation power when it comes to a small step size and large difference in boundaries. To avoid excessive iterations, the step size for weighting factors using grid search was selected to be 0.05 and 0.1.

**Figure 7:**
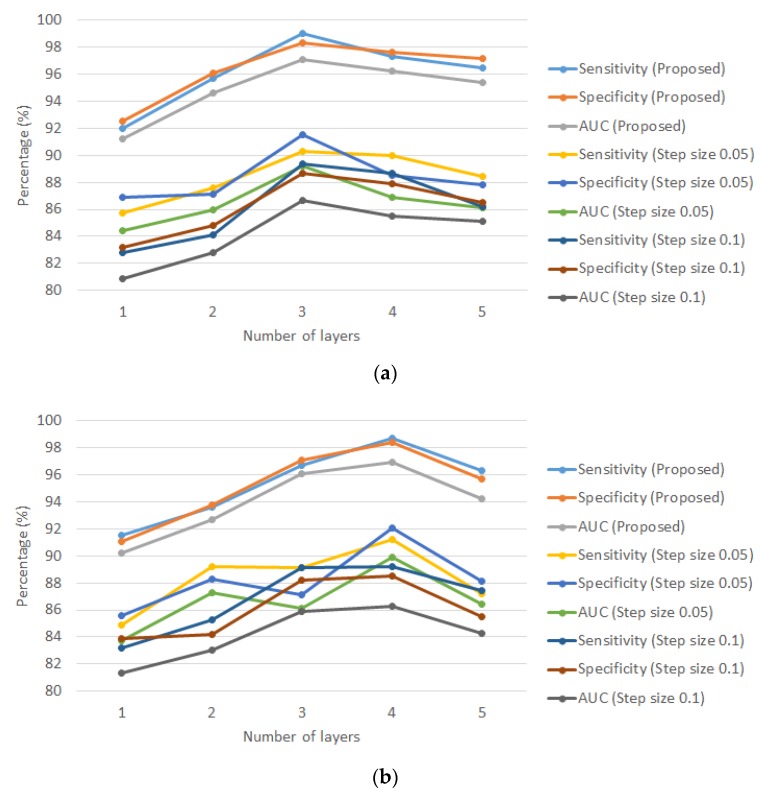
Average sensitivity, specificity and AUC of the proposed algorithm using MOGA and a traditional grid search with a step size of 0.05 and a step size of 0.1, versus the number of layers under a 10-fold cross-validation: (**a**) driver drowsiness recognition and (**b**) driver stress recognition.

Figure 7 shows the average sensitivity, specificity and AUC of the proposed algorithm and grid search method with a step size of 0.05 and 0.1, in driver drowsiness and stress recognition. The results were summarized as follows. For driver drowsiness recognition as in Figure 7a, the performance indicators of average sensitivity, specificity and AUC of the proposed algorithm were 99%, 98.3% and 97.1%. For the grid search approach with a step size of 0.05, the indicators were 90.3%, 91.5% and 89.2%. When the step size was doubled the indicators were 89.4%, 88.7% and 86.7%. Hence, the average improvement by MOGA was 8.64% and 11.2% comparing with grid search with a step size of 0.05 and 0.1 respectively.

When it comes to driver stress recognition, referring to Figure 7b, the proposed algorithm achieved 98.7%, 98.4% and 96.9%, the grid search approach with a step size of 0.05 achieved 91.2%, 92.1% and 88.9% and that for a step size of 0.1 was 89.2%, 88.5% and 86.3%. Thus, the average improvement by MOGA was 8.01% and 11.4% comparing with a grid search with a step size of 0.05 and 0.1 respectively.

It was concluded that the proposed algorithm outperformed the grid search method. All the methods agreed that the optimal numbers of deep layers for driver drowsiness and stress recognition were three and four respectively. A step size of 0.05 was better than that of 0.1 as the number of considered solutions was significantly increased. MOGA had better searching in the solution space whereas a grid search only bounds to limited solutions. One may argue that the step size can be further reduced so that all the possible solutions can be approximately analyzed, however, it requires extensive computation power, which is normally not a generic and feasible approach practically. Here, the proposed algorithm was a deep learning approach, which involved many optimization problems (optimal SVM).

### 3.3. Study on the Benefits of MKL

**Figure 8:**
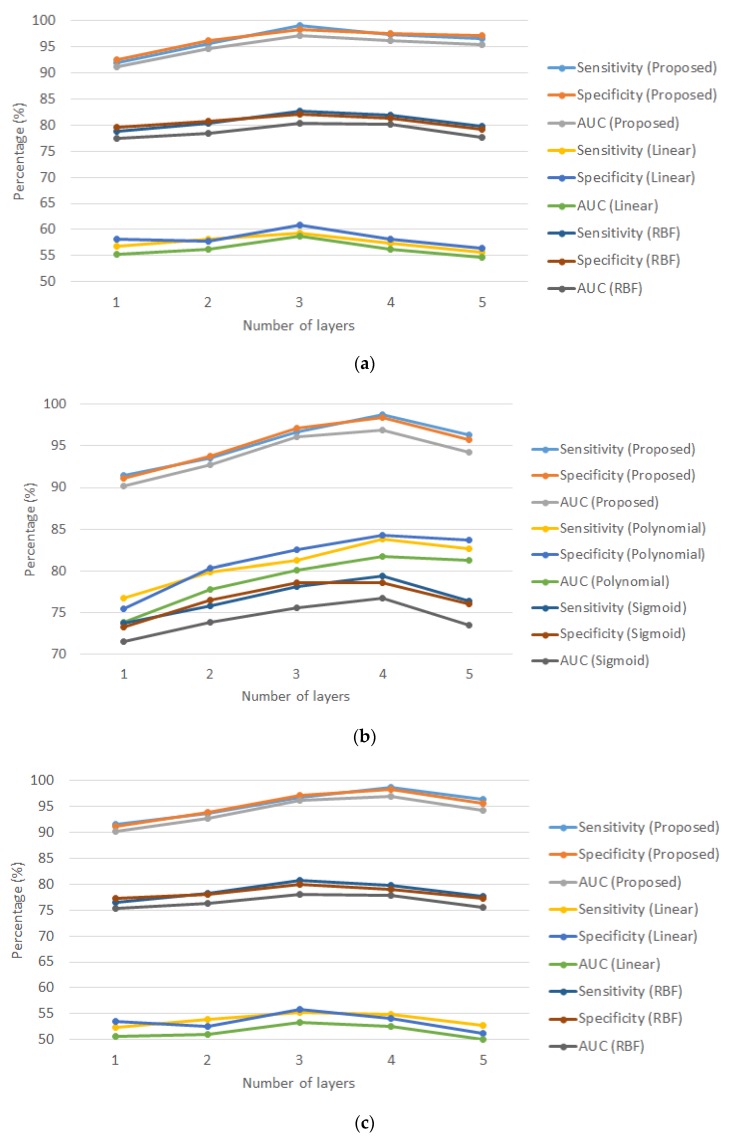

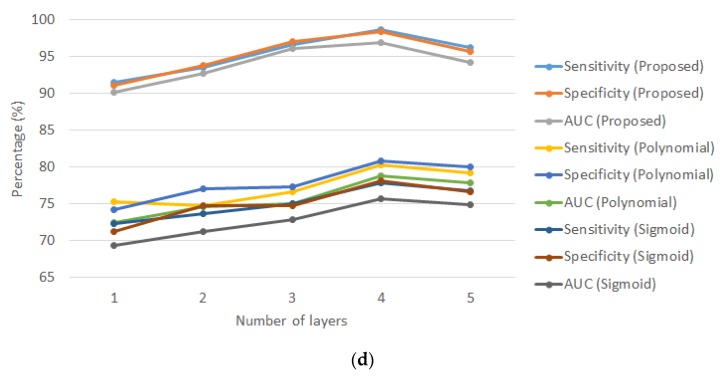
Average sensitivity, specificity and AUC of the proposed algorithm using multiple kernel learning (MKL) and typical single kernel versus number of layers under 10-fold cross-validation: (**a**) the MKL approach versus linear and radial basis function (RBF) kernels for driver drowsiness recognition; (**b**) the MKL approach versus polynomial and sigmoid kernels for driver drowsiness recognition; (**c**) the MKL approach versus linear and RBF kernels for driver stress recognition and (**d**) the MKL approach versus polynomial and sigmoid kernels for driver stress recognition.

To reveal the improvement of MKL, analysis is made between MKL and single typical kernel: linear kernel, RBF kernel, polynomial kernel and sigmoid kernel. Figure 8 shows the results of MKL, linear kernel, RBF kernel, polynomial kernel and sigmoid kernel for driver drowsiness and stress recognition. To better visualize the results, each subfigure compares MKL with two of the kernels.

Driver drowsiness recognition was analyzed based on Figure 8a,b. It can be concluded that the proposed algorithm using MKL significantly improved the performance indicators compared with all single typical kernel. Results between different kernel agreed with the optimal number of layers of three. The average performance improvement by the proposed algorithm was 64.6%, 20.1%, 17.7% and 25.2% compared to linear kernel, RBF kernel, polynomial kernel and sigmoid kernel, respectively.

The same finding was observed for driver stress recognition in Figure 8c,d. The optimal number of layers matches (which is four) in all approaches. On average, the performance improvement by the proposed algorithm was 82.0%, 24.3%, 22.5% and 26.9% respectively.

MKL combines the advantages and properties of various kernels so that the resultant kernel function outperforms any single typical kernel. One of the other key reasons is typical kernels are not customized to a specific application. Therefore, the proposed approach using MKL, which the weighting factors were optimally designed by MOGA, is a customized approach for designing the optimal kernel function for driver drowsiness and stress recognition.

### 3.4. Comparisons to Related Works

First, the comparison was carried out on driver drowsiness recognition. Table 2 summarizes the performance between proposed MOGA optimized D-MKL-SVM and existing works [16,17,18,19,20,21,22,23,24,25,26,27]. It summarizes the category of input signals, dataset, methodology, types of cross-validation and performance. Existing works may experience the following issues, which lead to lower reliability (and thus performance) when the methodology is implemented practically.

Generally, the first part of any methodology is the source of the input signal in which study in [37] analyzed the measurement reliability of several signals, ECG, EEG and video recording on real-world driving conditions (highway, urban and turning). EEG and a video recording may not be reliable input signals as only 85% and 59% of the time the acquired signals were with good signal quality. The severe signal distortion in the rest of the period could alter the validity of the foundation of the problem formulations, which could be considered as misleading input and thus output. Therefore, EEG and video-based approaches experience challenges in signal acquisition. As a result, the performance in related works [18,19,24,25,26,27] is reduced in a practical situation, which the methodology is governed by a maximum accuracy of 85% and 59% for an EEG-based and image-based approach.

It can be seen from Table 2 that some works [20,21,22,23,25,26,27] did not employ cross-validation. The bias training dataset (lack of generalization) may be selected to obtain high accuracy. In addition, many existing works [16,18,19,21,22,23,25,26,27] utilized simulation driving datasets. These were not convincing to reflect actual driver’s status.

The proposed MOGA optimized D-MKL-SVM algorithm utilizes the ECG signal, which provides a reliable measurement of stability. The real-world driving dataset and 10-fold cross-validation were adopted. Therefore, the performance of proposed work was more reliable and robust.

Consider driver stress recognition, Table 3 compares the performance between the proposed algorithm and related works [28,29,30,31,32,33,34,35], towards a category of input signals, dataset, methodology, types of cross-validation and performance. Similar to driver drowsiness recognition, related works in driver stress recognition experienced similar issues.

The EEG signal was adopted in [30], which the input signal has a measurement stability of 85% [37]. Some of the works [28,29,33] did not analyze the method using cross-validation. Data was collected using the simulated environment in [29,35]. It is noted that there is an unspecified dataset in [35]. To compare the rest of the existing works, the proposed algorithm outperforms [30,31,32,34] significantly.

As a result, the proposed MOGA optimized D-MKL-SVM outperforms existing works and serves as a generic approach for both driver drowsiness and stress recognition.

## 4. Conclusions

Owning to the fact that existing works on driver drowsiness and stress recognition have room for improvement, which suffer from the following concerns: (i) input signals of poor measurement stability, (ii) performance evaluation without cross-validation and (iii) collecting data using a simulated environment, this paper proposed a generic model using the multiple-objective genetic algorithm (MOGA) optimized deep multiple kernel learning support vector machine (D-MKL-SVM). For driver drowsiness recognition, the average sensitivity, specificity and AUC were 99%, 98.3% and 97.1% respectively. For driver stress recognition, the average sensitivity, specificity and AUC were 98.7%, 98.4% and 96.9% respectively. For performance evaluation, we firstly analyzed the importance of MOGA versus the traditional grid search method and MKL versus the single typical kernel. The proposed generic model achieved the highest accuracy for both driver drowsiness recognition and driver stress recognition compared to existing works.

The proposed approach can improve road safety as follows. Once drowsy events have been concluded, a sound could be emitted to awake drivers. In contrast to drowsiness recognition, stress recognition cannot directly prevent a stressed driving accident. The decision should come along with some relaxation techniques in order to lower the stress level of drivers. There are some measures to relieve stress while driving: (i) turn off disturbing noise, (ii) take deep breaths at stoplights, (iii) say what you see, (iv) try not to have long haul driving, (v) drive in slow and safe areas and (vi) embrace mindfulness. It is extremely important to reduce the stress level while driving. It not only helps at preventing accidents but also increases the happiness after driving, since stress can be accumulated as a snowball effect.

The future work will be suggested in two ways: (i) analyze the performance of proposed work with a real-world driving dataset, towards volunteers of different countries, so that the proposed algorithm can be adopted worldwide and (ii) test if the proposed generic model is applicable to cardiovascular diseases recognition because the proposed approach utilizes the ECG signal as an input, which is also the key input for cardiovascular diseases recognition.

## Figures and Tables

**Table 1:** Definition of classes and number of samples in driver drowsiness and stress dataset.

Dataset	Class	Number of Samples
Driver drowsiness dataset	Class 0: Awake stage	76,200
	Class 1: Drowsy stage 1	35,300
	Class 2: Drowsy stage 2	20,000
Driver stress dataset	Class 0: High stress level	19,300
	Class 1: Medium stress level	45,000
	Class 2: Low stress level	11,900

**Table 2:** Performance comparison between the proposed method and existing works for driver drowsiness recognition.

Work	Category	Dataset	Methodology	Cross-Validation	Performance
[16]	Biometric-signal-based (respiratory signal)	20 volunteers (simulated environment) Samples: 2246 awake; 1035 drowsy	Threshold-based approach by the tracking of the displacements of diaphragm, abdominal and rib cage	Leave-one-subject-out	Specificity: 96.6% Sensitivity: 90.3%
[17]	Biometric-signal-based (ECG signal)	18 volunteers (real-world driving environment) Samples: unknown	SVM using polynomial kernel using cross-correlation coefficient	10-fold cross-validation	Sensitivity: 77.4% Specificity: 76.5% Overall accuracy: 76.9%
[18]	Biometric-signal-based (EEG signal)	17 volunteers (simulated environment) Samples: 255 awake; 477 slightly drowsy; 167 moderate drowsy; 98 significant drowsy; 20 extremely drowsy	SVM using RBF kernel using RBP (α) and movement power	Leave-one-subject-out	Overall accuracy: 93.7%
[19]	Biometric-signal-based (EEG signal)	16 volunteers (simulated environment) Samples: unknown	LSTM using spectral entropy and instantaneous frequency	10-fold cross-validation	Overall accuracy: 94.3%
[20]	Vehicle-based (steering wheel angle)	6 volunteers (real-world driving environment) Samples: 92 awake; 99 drowsy	Threshold-based approach by analyzing steering wheel angle	No	Accuracy: 78.0%
[21]	Vehicle-based (steering wheel angle)	10 volunteers (simulated environment) Samples: Total 7020	Multilevel ordered logit model	No	Accuracy: 72.9%
[22]	Vehicle-based (steering wheel angle, pedal input, vehicle speed and acceleration)	72 volunteers (simulated environment) Samples: 840 awake; 21 drowsy	Dynamic Bayesian Network algorithm	No	Specificity: 85% AUC: 77%
[23]	Vehicle-based (deviation from the current lane)	Unknown number of volunteers (simulated environment) Samples: 4000 awake; 4000 drowsy	Exponentially weighted moving average	No	Sensitivity: 76% Accuracy: 86%
[24]	Image-based (image of driver’s head)	30 volunteers (real-world driving environment) Samples: 11568 awake; 4224 moderate drowsy; 4560 severe drowsy	Deep belief network	10-fold cross-validation	Average accuracy: 96.7%
[25]	Image-based (eyes)	Unknown number of volunteers (simulated environment) Samples: 2500 images	Threshold-based approach by analyzing eye blinking frequency	No	Accuracy: 89%
[26]	Image-based (eyes)	15 volunteers (simulated environment) Samples: 1068 images in total	Fusion and reasoning method including head and shoulder detection, face detection based on front view and oblique view analysis, eye detection	No	Average accuracy: 90.1%
[27]	Image-based (yawns and eyes)	15 volunteers (simulated environment) Samples: unknown	Threshold-based approach using second-order blind identification algorithm	No	Accuracy: From 27.2% to 95.3% to under different scenarios
Proposed algorithm	Biometric-signal-based	126 volunteers (real-world driving environment) Samples: 76,200 awake; 35,300 sleep stage 1, 20,000 sleep stage 2	MOGA optimized D-MKL-SVM with cross-correlation and convolution coefficients	10-fold cross-validation	Sensitivity: 99% Specificity: 98.3% AUC: 97.1%

Area under the curve (AUC); Receiver operating characteristic (ROC); Deep multiple kernel learning support vector machine (D-MKL-SVM); Electrocardiogram (ECG); Electroencephalography (EEG); Long short-term memory (LSTM); Multiple-objective genetic algorithm (MOGA); Radial basis function (RBF); Relative band power (RBP); Support vector machine (SVM).

**Table 3:** Performance comparison between the proposed method and existing works for driver stress recognition.

Work	Category	Dataset	Methodology	Cross-Validation	Performance
[28]	Biometric-signal-based (HRV)	22 volunteers (real-world driving environment) Samples: Unknown	Wilcoxon Signed rank test, *t*-test and ANOVA	No	No (statistical analysis between HRV and stress level)
[29]	Biometric-signal-based (EDA SPR)	15 volunteers (simulated environment) Samples: 510	Adaptive filtering and spike detection	No	Accuracy: 83.9%
[30]	Biometric-signal-based (Skin conductance and EEG)	30 volunteers (real-world driving environment) Samples: 713 normal; 430 low anger; 315 medium anger; 161 high anger	Incremental association Markov blanket and least square SVM	10-fold cross-validation	Accuracy: 82.2%
[31]	Biometric-signal-based (HRV and PPG)	21 volunteers (real-world driving environment) Samples: Unknown	Ensemble learning of kNN, DT and LDA	10-fold cross-validation	Accuracy: 86.9%
[32]	Biometric-signal-based (GSR), HRV and respiration)	18 volunteers (real-world driving environment) Samples: 588 high stress level; 588 medium stress level; 588 low stress level	SVM and ELM	Leave-one-subject-out	SVM Sensitivity: 88.5% Specificity: 94.2% ELM Sensitivity: 88.2% Specificity: 94.1%
[33]	Vehicle-based (steering wheel angle)	8 volunteers (simulated environment) Samples: 2154 normal; 2287 stress	SVM	No	Accuracy: 82.5%
[34]	Vehicle-based (steering wheel angle and road shape)	4 volunteers (real-world driving environment) Samples: 220	Multilayer perceptron	10-fold cross-validation	Accuracy: 46.9%
[35]	Speech-based (speech and GSR)	N/A	SVM	10-fold cross-validation	92.4%
Proposed algorithm	Biometric-signal-based	18 volunteers (real-world driving environment) Samples: 19,300 high stress level; 45,000 medium stress level; 11,900 low stress level	MOGA optimized D-MKL-SVM with cross-correlation and convolution coefficients	10-fold cross-validation	Sensitivity: 98.7% Specificity: 98.4% AUC: 96.9%

Analysis of variance (ANOVA); Decision tree (DT); Electrodermal activity (EDA); Extreme learning machine (ELM); Galvanic skin response (GSR); Heart rate variability (HRV); k-nearest neighbor (kNN); Linear discriminant analysis (LDA); Photoplethysmogram (PPG); Signal potential response (SPR).
